# Synthetic biology routes to new and extinct natural products

**DOI:** 10.1039/d5cb00047e

**Published:** 2025-03-21

**Authors:** Thomas J. Simpson

**Affiliations:** a School of Chemistry, University of Bristol Bristol BS8 1TS UK tom.simpson@bristol.ac.uk

## Abstract

Recent developments in genome sequencing and genetic engineering have revolutionised elucidation of biosynthetic pathways in bacteria and fungi and allowed production of new natural products and engineered strains with optimised production of new and/or preferred metabolites. The clinically important antibiotic mupirocin is a mixture of closely related pseudomonic acids produced by *Pseudomonas fluorescens via* a *trans*-AT modular PKS. Extensive gene knock-out experiments have led to the isolation of a plethora of new metabolites: both biosynthetic intermediates and shunt products. Parallel experiments, along with swapping of biosynthetic genes, with a *Pseudoalteromonas* sp. which produces the closely related thiomarinols give similar results and many new products. A genetically engineered strain of *P. fluorescens* produces high titres of a single pseudomonic acid with improved stability and antibiotic properties. Tenellin and bassianin are insecticidal fungal metabolites produced by *Beauvaria* species *via* multi-domain PKS-NRPSs. Heterologous expression in *Aspergillus oryzae* of hybrid systems produced by domain swapping between the two biosynthetic gene clusters produce many new metabolites in high yields and reveal the key elements in control of polyketide chain length and methylation, showing the potential for combinatorial biosynthesis of these and related metabolites. *Cryptosporiopsis* sp. 8999 produces three related dimeric xanthones. Gene knock-outs allow elucidation of the full biosynthetic pathway, isolation of the monomeric precursor and engineering of a strain producing only the major component of the wild-type mixture.

## Abstract 2

Developments in genome sequencing and genetic engineering in bacteria and fungi and allowed production of new natural products and engineered strains with optimised production of new and/or preferred metabolites.

## Graphical abstract

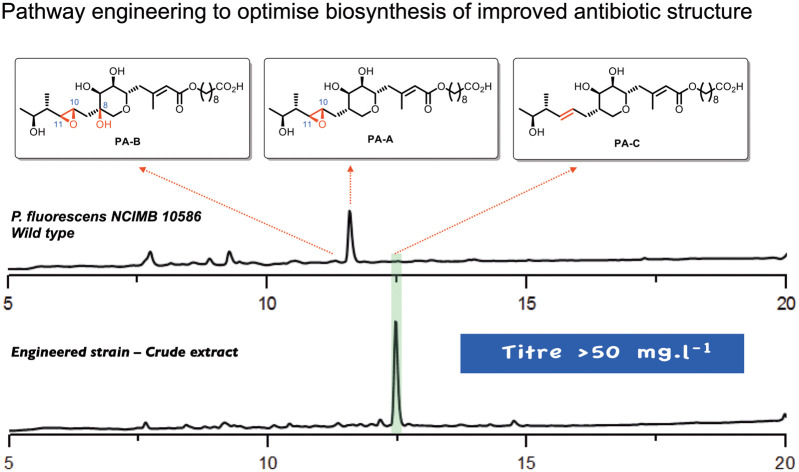

## Introduction

Natural products isolated from plants, marine organisms, fungi and bacteria have long been a major source of bioactive compounds with a wide range of biological activities making them a highly important source of medicines and agrochemicals.^1^ The combination of their intriguing structures and interesting properties have made them important targets for synthetic and medicinal chemists.^2^ Apart from the technical and intellectual challenges, this is often justified by the limited amounts sometimes available from the natural sources, and the development of synthetic routes that would allow access to structural variations for structure activity studies and optimisation of biological activities and other desirable properties, *e.g.*, stability and solubility.

In the same way that they have attracted the attention of the synthetic community, studies of their biosynthesis – the metabolic pathways by which they are elaborated in their producing organisms – has long excited the biosynthetic community. These studies have taken many forms – initially isolation of structurally related compounds which might be intermediates or side (shunt) products that may provide evidence of the biosynthetic sequence.^3^ These were soon augmented by the increasing availability and use of radioactive isotopes (carbon-14 and tritium) which allowed postulated early or advanced intermediates to be selectively labelled and their incorporation into the final metabolite determined.^4^ This again often necessitated development of sophisticated synthetic methods to strategically place the isotopes in critical positions in the molecule to be incorporated, and elaborate degradation routes to establish that the label had been incorporated site specifically and not randomly. Classical biosynthetic studies on *e.g.* alkaloid biosynthesis in higher plants relied heavily on this methodology. These, however, were typified by often extremely low levels of incorporations of precursors. The advent of the ready availability of stable isotopes, ^13^C, ^2^H, ^15^N, ^18^O and the contemporaneous development of NMR and MS methods for structure elucidation and specific location of labels (and multi-labelling) largely superseded radioisotopes especially in microorganisms where the necessary higher levels of incorporation were more readily achieved. This led to a huge advance in the understanding and elucidation of the details of biosynthetic pathways.^5^ These methods obviated the need for complicated degradations and paralleled the increasing dominance of NMR methods for natural product structure elucidation.

In sharp contrast to primary metabolism, and apart from a few heroic efforts in higher plants,^6^ relatively little was done on the detailed enzymology of secondary metabolic pathways. The advent of molecular genetics and the realisation that all, or at least most, of the genes involved in natural product biosynthesis were normally “clustered” on contiguous stretches of DNA in fungi and bacteria was the next and arguably most significant advance in biosynthetic studies.^7^ This was further augmented by the increasing ease of whole genome sequencing, computational methods of analysis, *e.g.* anti-SMASH^8^ allowing assignment of catalytic function to gene products, and the ability to carry out genetic manipulation. These include overexpression of genes allowing access to biosynthetic enzymes, selective gene deletions to isolate/trap intermediates, methods for heterologous expression of genes, and addition and combination of genes from related pathways. In this review we shall illustrate some applications of these methods drawn from our own work on bacterial and fungal polyketides to the production of novel – new to nature or not so new to nature – metabolites, the production of “improved” structures and generation of (multiple) analogues and structural variations not readily amenable to synthetic methodologies. In addition, we can develop rational improvements of overall titres, and engineer strains which give selective production of only a desired compound rather than mixtures which may be problematic to separate.

## Mupirocin/thiomarinol

Mupirocin is a clinically important antibiotic, produced by the bacterium *Pseudomonas fluorescens*.^9^ It is mainly used for topical treatments such as acne and as a nasal spray for pre-op prophylactic treatment against MRSA, multiple resistant *Staphylococcus aureus*.^10^ It acts by inhibiting isoluecyl-tRNA synthetase, but its use has been limited by its inherent instability and susceptibility to hydrolysis. It consists of a mixture of pseudomonic acids (PAs). The main constituent is PA-A (*ca.* 90%) 1, PA-B (7%) 2 and PA-C < (2%) 3. A main cause of its instability is the intramolecular addition of the 7-hydroxyl onto either C-10 or C-11 of the 10,11-epoxide to give cyclic ethers which are inactive. Biogenetic analysis based on precedent and logic would suggest that initially formed PA-C is epoxidized to give PA-A followed by further hydroxylation at C-8 to give PA-B. We thus postulated that if the gene controlling epoxidation could be identified, gene knock-out would give PA-C, which lacking the epoxide would be more stable and thus should be an improved and more widely useable antibiotic. An alternative strategy to achieve the same aim uses the closely related thiomarinol A 4. Thiomarinols are a group of related antibiotics produced by a marine bacterium *Pseudoalteromonas* SANK73390.^11,12^ The structures of the major metabolites, thiomarinol A and pseudomonic acid A (PA-A), differ by the presence of a 10,11-alkene *versus* an epoxide, an additional 4-hydroxyl, an 8-hydroxyoctanoic acid rather than 9-hydroxynonanoic acid side chain, attached to an additional pyrrothine moiety *via* an amide linkage to the hydroxy acid moiety.

**Fig. 1:**
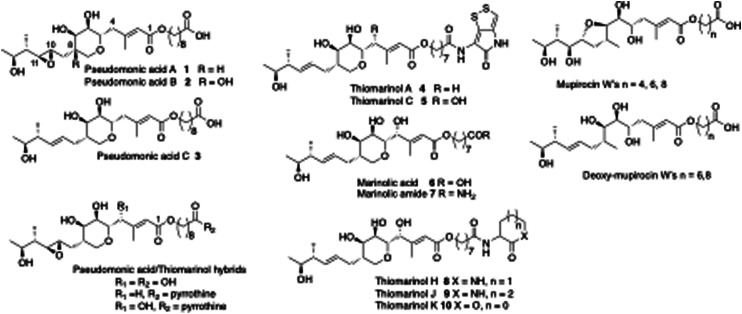
Selected pseudomonic acid, thiomarinol and mupirocin metabolites.

The mupirocin biosynthetic gene cluster (BGC), designated *mup*, was identified in 2003 (Fig. 1A).^13^ It shows an unprecedented complexity, with a series of PKS and related modules and an unexpectedly large set of tailoring genes, itself suggestive of an unusually complex biosynthetic pathway. Mutational analysis showed that all the genes were essential for mupirocin production.^14^

**Scheme 1:**
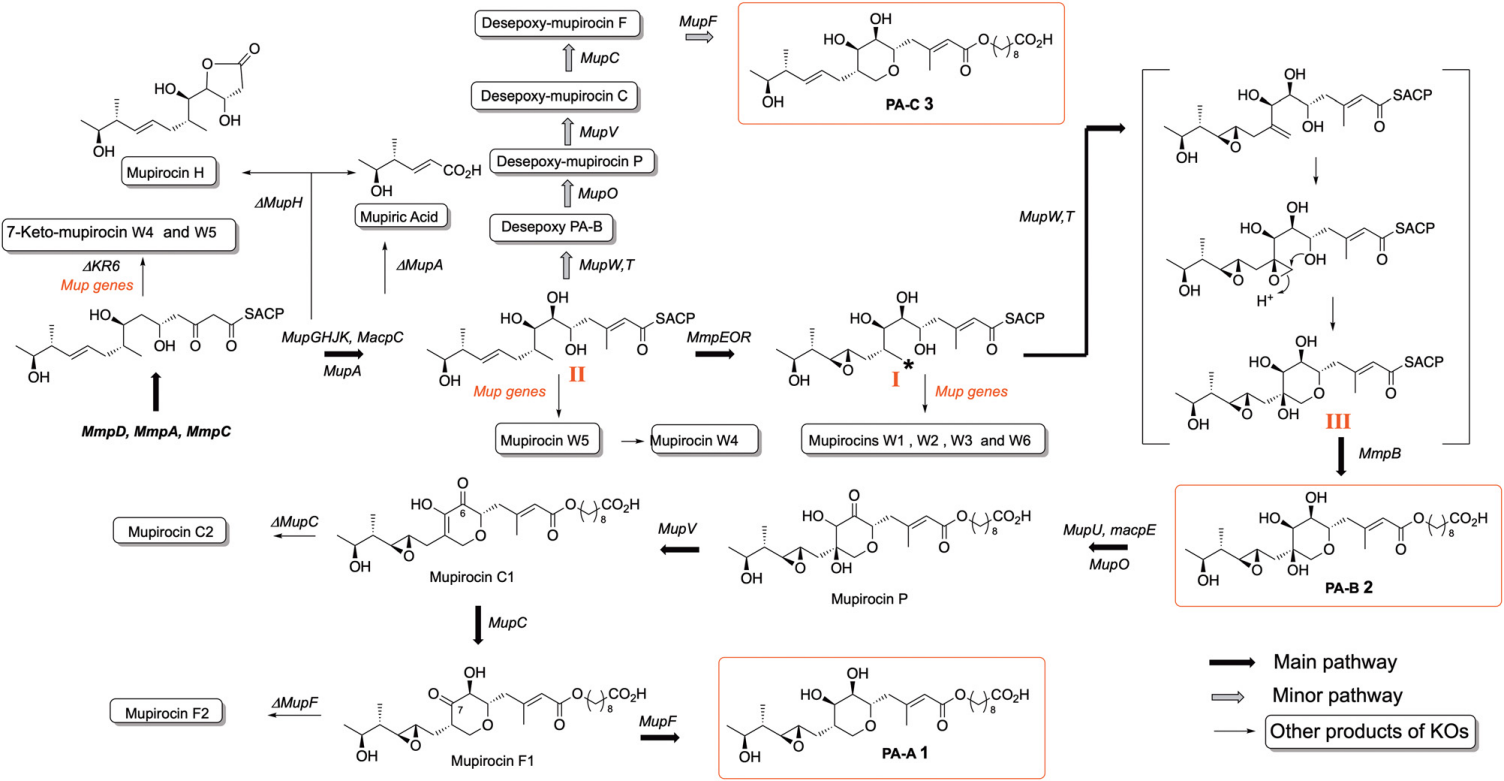
Mupirocin biosynthetic pathway and products of gene KOs.

**Fig. 2:**
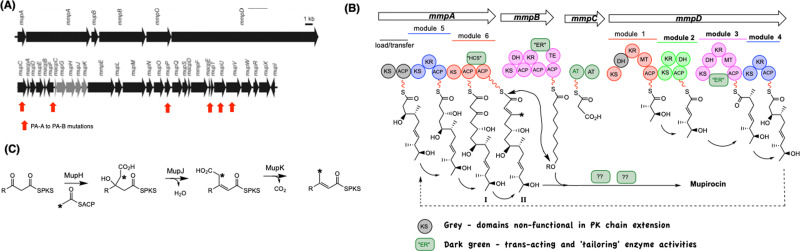
(A) Summary of the mupirocin biosynthetic gene cluster. The HCS cassette responsible for β-branching is shown in grey. (B) Formalized scheme for the biosynthesis of monic acid and its elaboration to mupirocin. “Grey” domains are inactive or non-functional in chain elongation. Tailoring proteins acting *in trans* are indicated as elongated domains. (C) Scheme for incorporation of a C-15 methyl group by HCS cassette.

However, mutation of each of a series of apparently unrelated genes all resulted in a surprising total switch from production of mainly PA-A to give high titres of exclusively PA-B, indicating that PA-B must be a precursor to PA-A.^15^ This conundrum was eventually solved by the pathways shown in Scheme 1^16^ in which a methionine derived methyl (*) in a linear polyketide intermediate I is converted by dehydrogenation, epoxidation and an intriguing anti-Baldwin cyclisation to form III with the THP core essential for activity.^17^ After MmpB mediated production and addition^18^ of the fatty acid to give PA-B, subsequent steps analogous to those found in deoxysugar biosynthesis then remove the 8-hydroxyl from PA-B to give PA-A.^16^ All the intermediates shown between PA-B and PA-A have been isolated and characterised from the appropriate mutant KO strains (Fig. 2A). Intriguingly it is only when the last step of formation of PA-A takes place that full antibiotic activity is revealed.

**Fig. 3:**
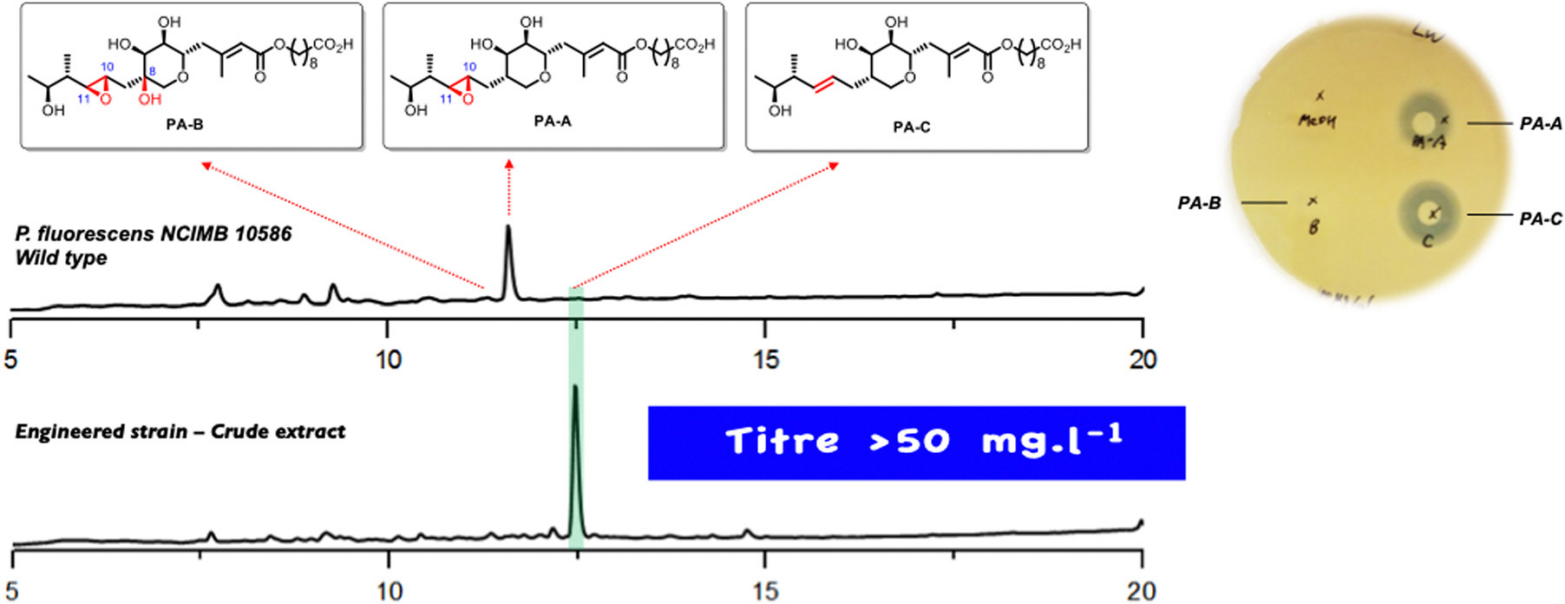
HPLC trace of the *P. fluorescens* wild type and *mmpE*Δ*OR* mutant cultured in baffled flasks. Plate assay of PAs against MRSA.

In a minor pathway the non-epoxidised analogue II of linear intermediate I follows exactly the same sequence to produce the minor PA-C product, again all the intermediates arising from KOs of *mupF*, *C*, *V* and *O* have been isolated in good yields.^19^ With this information to hand it was postulated that identifying and blocking the 10,11 epoxidation should divert the biosynthesis down the minor pathway to give the desired PA-C as the main product. The gene responsible was identified as an oxidase domain in the multidomain protein encoded by *mmpE* and KO of the oxidase blocked PA-A production and did indeed give PA-C albeit in low titre. However, minor modifications of the fermentation conditions gave a very high producing strain with PA-C as the sole main product (Fig. 3).^20^ As summarised in Scheme 1, systematic gene knock-out experiments have resulted in isolation of a very large number of linear and ring containing mupirocin analogues, but only PA-A and PA-C show significant antibiotic activity.

**Fig. 4:**
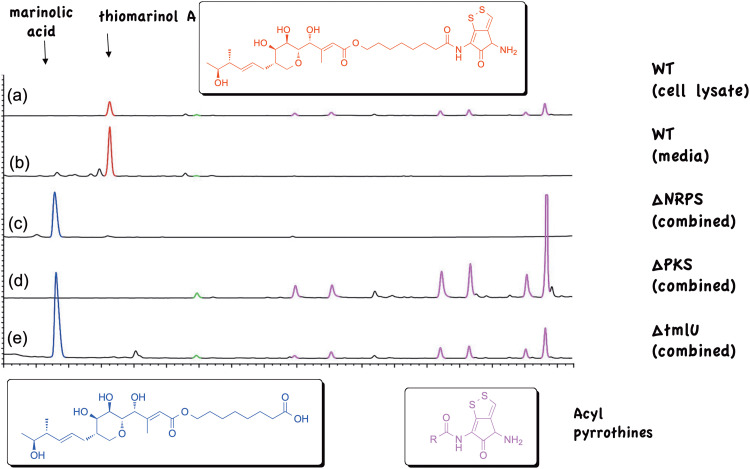
Products of wild type and mutant strains of Pseudoalteromonas SANK73390.

The thiomarinols are structurally very similar to pseudomonic acids and are also powerful anti-MRSA antibiotics but were not developed clinically due to toxicity issues. The strain also produces a series of xenorhabdins, pyrrothines acylated with a range of long chain (C_8_–C_16_) saturated and unsaturated fatty acids (Fig. 4a).^21^ These acylpyrrothines show antibiotic activities in their own right and are thought to inhibit RNA polymerase. The BGC (*tml*) was identified *via* genome sequencing^22^ and contains, as expected, *trans*-AT PKS and associated tailoring genes with high homology to the mupirocin (*mup*) cluster, along with genes (*holA-H*) encoding a non-ribosomal peptide synthetase (NRPS) linked to a set of tailoring enzymes, similar to those shown to control holomycin (acetyl-pyrrothine) biosynthesis in *Streptomyces clavuligerus.*^23^ An internal segment of the NRPS gene (*holA*) was used to generate a ΔNRPS strain.^24^ This abolished biosynthesis of both thiomarinols and the acylpyrrothines and produced mainly a PA-C analogue, marinolic acid 6 lacking the pyrrothine moiety (Fig. 4c), and two minor analogues with truncated (C_6_ and C_4_) fatty acid chains and marinolic amide 7. A ΔPKS mutant produced no thiomarinols but retained the xenorhabdins (Fig. 4d). Finally an in frame deletion of *tmlU* a putative acyl CoA synthase^25^ resulted in a strain (Δ*tmlU*) which did not produce thiomarinols but produced both marinolic acid and the xenorhabdins (Fig. 4e).

A series of mutasynthesis experiments was carried out with these mutants.^26^ When pseudomonic acid A was fed to the ΔPKS mutant, pyrrothine addition and/or 4-hydroxylation occurred to give its corresponding thiomarinol A and thiomarinol C analogues and to 4-hydroxy-PA-A, lacking addition of pyrrothine (Fig. 1). Thus TmlU and the 4-hydroxylase are able to accept close analogues of thiomarinol. PA-C was similarly metabolised. In contrast, feeding PA-B or desepoxy-PA-B simply led to pyrrothine addition, and no 4-hydroxylated metabolites were detected. Hence it is apparent that the presence of an 8-hydroxy group inhibits 4-hydroxylation. A comparison of the mupirocin and thiomarinol gene clusters to identify genes that lack paralogues in the mupirocin cluster and therefore might encode the 4-hydroxylase identified TmuB as an αKG-dependent dioxygenase.^27^ The *tmuB* coding region was inserted into wild-type *P. fluorescens via* the broad-host-range vector pJH10. HPLC analysis of the culture supernatant after *tmuB* expression showed a main metabolite more polar than PA-A, confirmed to be 4-hydroxy-PA-A. Similar transformation of the PA-C producing mutant gave 4-hydroxy-PA-C, but mutant strains which produced PA-B, showed no conversion to the 4-hydroxylated analogue, consistent with the feeding experiments to the ΔPKS mutant described above. Kinetic experiments with purified TmuB indictaed that PA-B is a competitive inhibitor of the enzyme. Deletion of *tmlF*, similar to *mupF* which catalyses the last step to PA-A, led to isolation of new thiomarinol metabolites analogous to PA-B and mupirocins C and F, and their deoxy analogues (Scheme 1) all containing the 4-hydroxy and pyrrothine. As might have been anticipted, however, 8-hydroxy-thiomarinol C fails to gain the 4-hydroxyl.

The mutasynthetic incorporation of alternative amines in the Δ*NRPS* mutant was also examined. The isolation of thiomarinol H 8 was reported from a related organism, now unavailable, *Altermonas rava*,^28^ but not previously from SANK73390, prompted us to feed anhydro-ornithine and related amines. Ornithine itself has no effect on SANK73390 which may have lost its ability to cyclise ornithine to its anhydro-form. When this was fed, both thiomarinol H and thiomarinol A were produced, indicating that anhydro-ornithine can compete with pyrrothine as a coupling partner in thiomarinol production. Similarly anhydrolysine gave a small amount of the corresponding 7-membered lactam, a “new to nature” analogue, which was named thiomarinol J 9. In contrast α-aminobutyrolactone was not incorporated but the linear homoserine did yield the novel 5-membered lactone analogue, by analogy called thiomarinol K 10.

These studies show the value of mutagenesis in producing strains with enhanced and selective production of metabolites with optimised activities. Along with parallel mutasynthesis a very wide range of novel metabolites can be obtained. Bioassays of most of the novel compounds indicate that PA-A and PA-C, their 4-hydroxy and pyrrothine analogues, thiomarinol A, and marinolic acid all show similar levels of activity against MRSA and a range of other bacteria, but PA-B and all compounds lacking the mature dihydroxy-THP ring display only limited activities. The availibility of compoundswith truncated chain length fatty acid moieties also confirmed the need for at least a C_6_ fatty acid chain for activity, in agreement with X-ray studies of PA-A bound to isoleucyl tRNA synthetase.^10^

## Tenellin/bassianin PKS-NRPS domain swaps

**Scheme 2:**
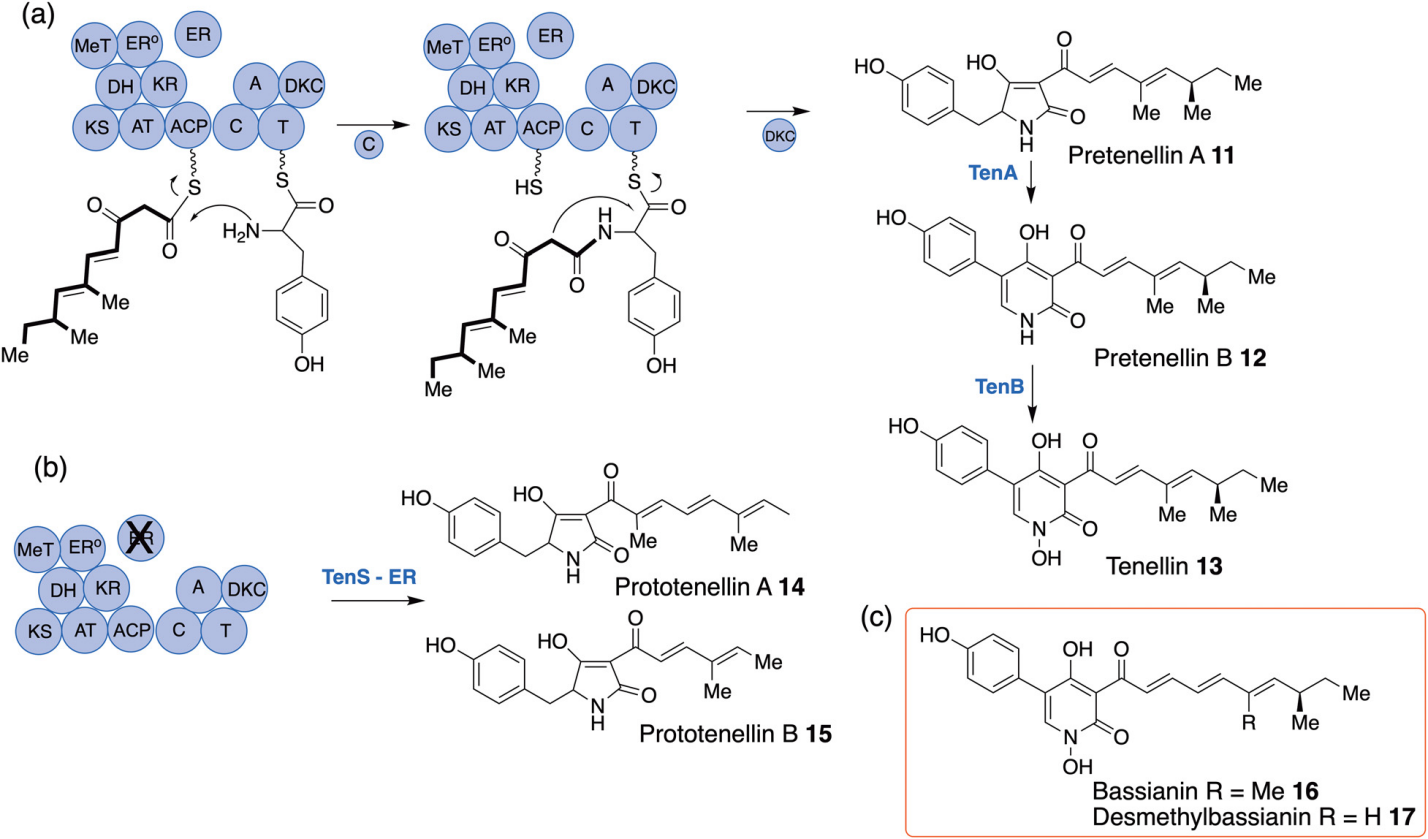
(a) Tenellin biosynthetic pathway. (b) Products of TenS in absence of *in trans* ER, TenC. (c) Bassianin metabolites.

The fungal metabolites tenellin 13 and bassianin 16 (Scheme 2) are fungal metabolites produced somewhat confusingly from the insect pathogenic fungus *Beauvaria bassaina* and *Bassiana tenacellus* respectively.^29^ They are produced *via* fungal polyketide non-ribosomal peptide synthetase (PKS-NRPS) proteins, first described for biosynthesis of the mycotoxin fusarin C in *Fusarium moniliforme*^30^ but now known to be widespread. Tenellin, pentaketide, and bassianin, hexaketide, differ only in the chain length of the polyketide moiety, bassianin containing one extra *trans*-olefinic unit. The tenellin BGC is relatively simple, consisting of genes encoding the PKS-NRPS (TenS), an enoyl reductase (TenC) which works in tandem with TenS to produce the first isolable intermediate, pretenellin A 11, and two P_450_ enzymes, TenA and TenB.^31^ The tenellin PKS belongs to the highly-reducing Type 1 iterative family (hr-PKS)^32^ and catalyses formation of a bis-*C*-methylated pentaketide with two methyl branches being introduced by a *C*-methyl transferase (MeT) domain, the *trans* olefins by the keto-reductase (KR) and dehydratase (DH) domains and the saturated bond by both of these working in concert with the *in trans* enoyl reductase (ER) (Scheme 2a). The polyketide is bound to an ACP before being condensed with tyrosine loaded onto the thiolation (T) domain of the NRPS by the adenylation (A) domain as an amide, bond formation catalysed by the condensing (C) domain. The PKS-NRPS terminates in a “Dieckmann condensation” domain (DKC) which effects cyclisation and release from the NRPS as the tetramic acid, pretenellin A 11. The first P_450_ (TenA) then catalyses an oxidative ring expansion of the tetramic acid to a pyridone, pretenellin B 12 and the second (TenB) an *N*-hydroxylation to tenellin 13.^33^ Heterologous expression of all 4 genes in *Aspergillus oryzae* results in very high levels of tenellin production, 6 times higher than fermentation of *B. bassiana* itself.^34^ Omitting *tenB* from the expression system results in production of pyridone, pretenellin B and omission of both *tenA* and *tenB* and gives high yields of the tetramic acid, pretenellin A.^35^ Interestingly, expression of TenS only without the *trans*-acting ER (TenC, Scheme 2b) gives a rather complex mixture with two isolable main tetramic acids, prototenellins A and B 14 and 15 varying in chain length, number and positioning of *C*-methylation suggesting that physical association of the ER with the PKS plays a key role in fidelity of polyketide assembly.^36^

Bassianin 16 contains a dimethylated-hexaketide in contrast to the dimethylated-pentaketide in tenellin. To investigate what element controls chain length in these systems, we planned parallel experiments with the bassianin gene cluster. However, these were frustrated by the inability to locate a bassianin producing *Beauvaria* strain, the original producing strain no longer being available. Thus, bassianin is effectively an “extinct” metabolite. However, after extensive screening of some 30 *Beauvaria* strains,^36^ one was found which produced a novel monomethylated derivative, desmethybassianin 17, simply lacking introduction of the second *C*-methylation step in bassianin (Scheme 2c), thus, allowing us to study a further control element – *C*-methylation. The genome was sequenced and the *dmb* BGC identified: this had very high homology to the ten cluster. Parallel gene expression experiments then gave exactly analogous results to those in tenellin.^36^

**Fig. 5:**
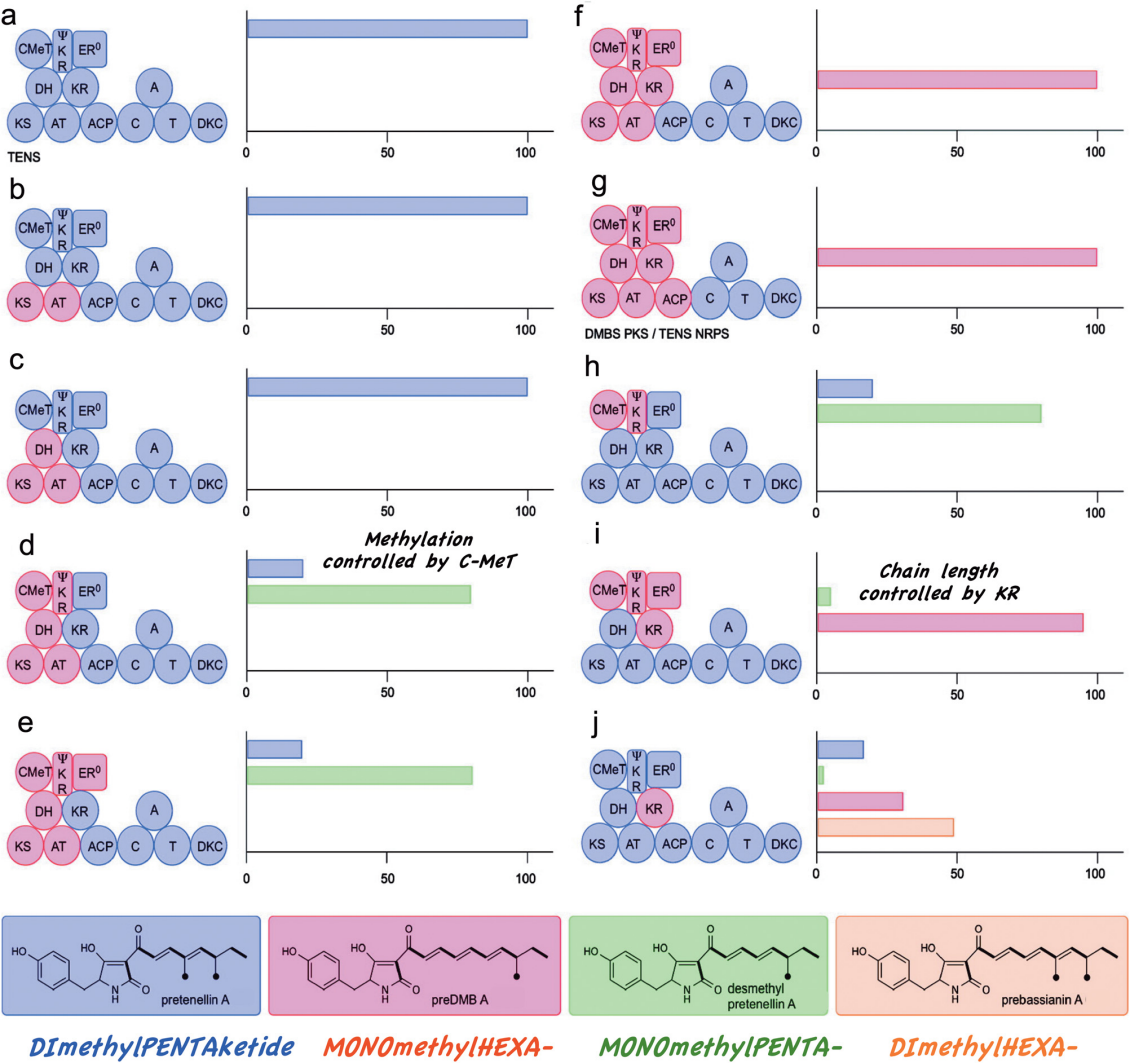
Domain architectures of chimeric PKS-NRPS constructs and relative titres of resulting metabolites. Co-expression with either the Tens or Dmb *in trans* ER domains were equally effective.

A series of gene constructs were then prepared where the DNA encoding domains in the tenellin PKS moiety (blue) were successively replaced by the corresponding desmethylbassianin (pink) domains.^37^ The overall aim being to elucidate what controlled the differing aspects of polyketide assembly: chain length, degree of methylation, degree of reduction, *etc.* This resulted in a number of “new to nature” compounds being produced again in high titres (Fig. 5). Substitution of the first few domains had no observable effect until the Dmb *C*-MeT domain was included. The major product of this construct (Fig. 5d) was found to be a novel monomethylated-pentaketide containing metabolite, desmethylpretenellin 18 indicating not surprisingly, that the *C*-Met domain influences the number of methylation steps. Further inclusion of the Dmb KR domain (Fig. 5f and g), gave a complete switch to produce only the monomethylated-hexaketide, predesmethylbassianin A 19, indicating, somewhat surprisingly, that it is the KR domain that primarily determines chain length.

**Scheme 3:**
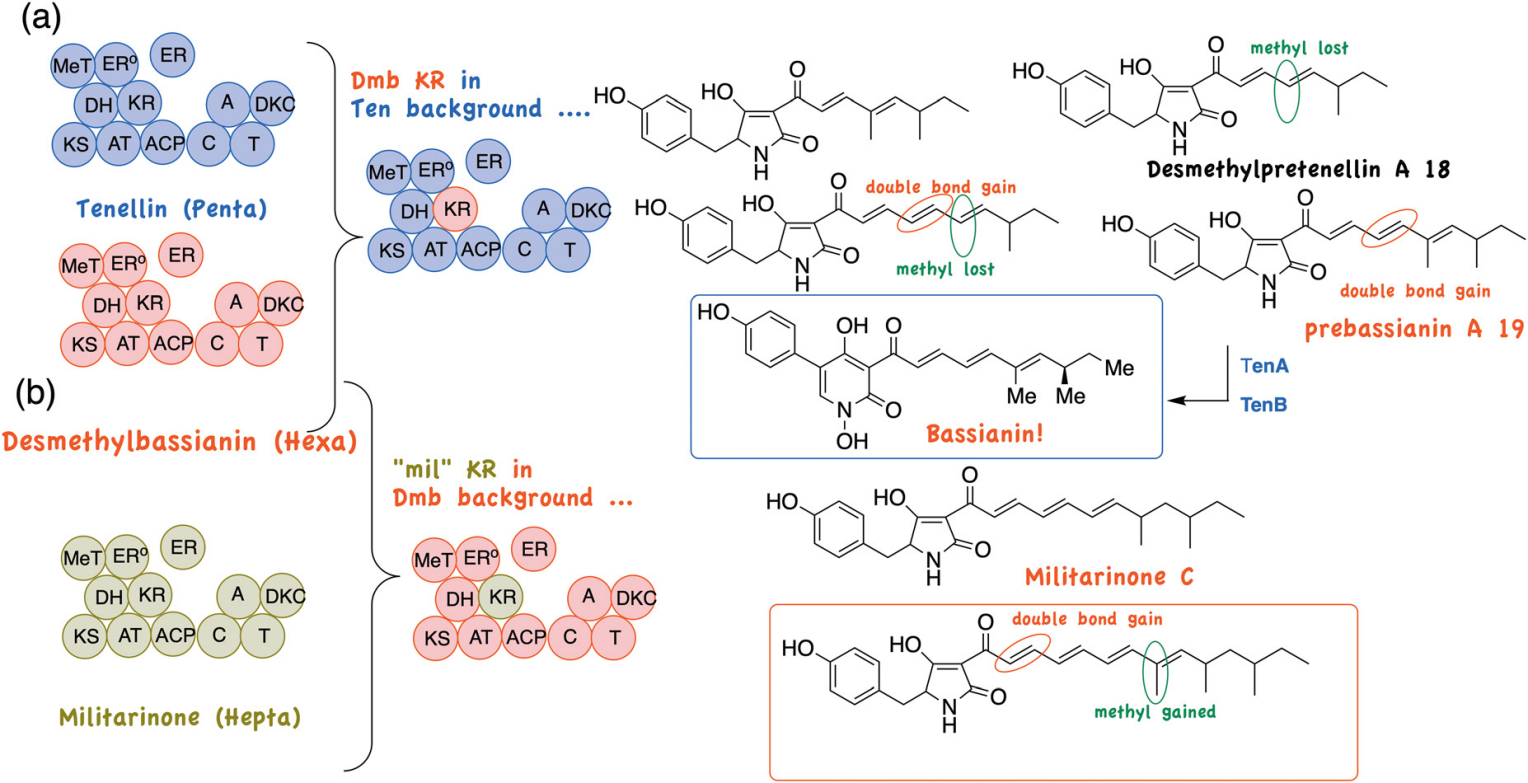
Single KR domain replacements, (a) DMB KR in a TenS background and (b) Mil KR in a Dmb backgound.

Interestingly in heterologous PKS constructs, the KR domain has a small effect on the fidelity of MeT catalysed methylation and *vice versa*. This was verified by preparing single domain replacement constructs in which only the TenS MeT was included in an otherwise Dmb background (Fig. 5h) and one in which the Dmb KR domain was included in an otherwise TenS background (Fig. 5j and Scheme 3a). This gave a mixture of 4 compounds, including minor amounts of pretenellin and desmethylpretenellin indicating some loss of fidelity in these heterologous constructs. As expected total hexaketides dominated (80%) over pentaketides (20%) – KR control, and dimethylated products (66%) over monomethylated (34%) – MeT control. Gratifyingly of the two hexaketide derived products the major component was “prebassianin A” 19 the precursor to the “extinct” bassianin. Co-expression of tenA and tenB with this construct gave another four products, the major being the long lost bassianin. We have called such “raised from the dead” metabolites “Lazarus compounds”! Thiomarinol H above can be similarly classified. The principle can be extended to other more heterologous systems. Militarinone, a tetramic acid metabolite of *Paecilomyces militaris* contains a bis-*C*-methylated heptaketide.^37^ Insertion of the militarinone KR into a Dmb background (Scheme 3b) now gives a trimethylated octaketide as a main product.

**Fig. 6:**
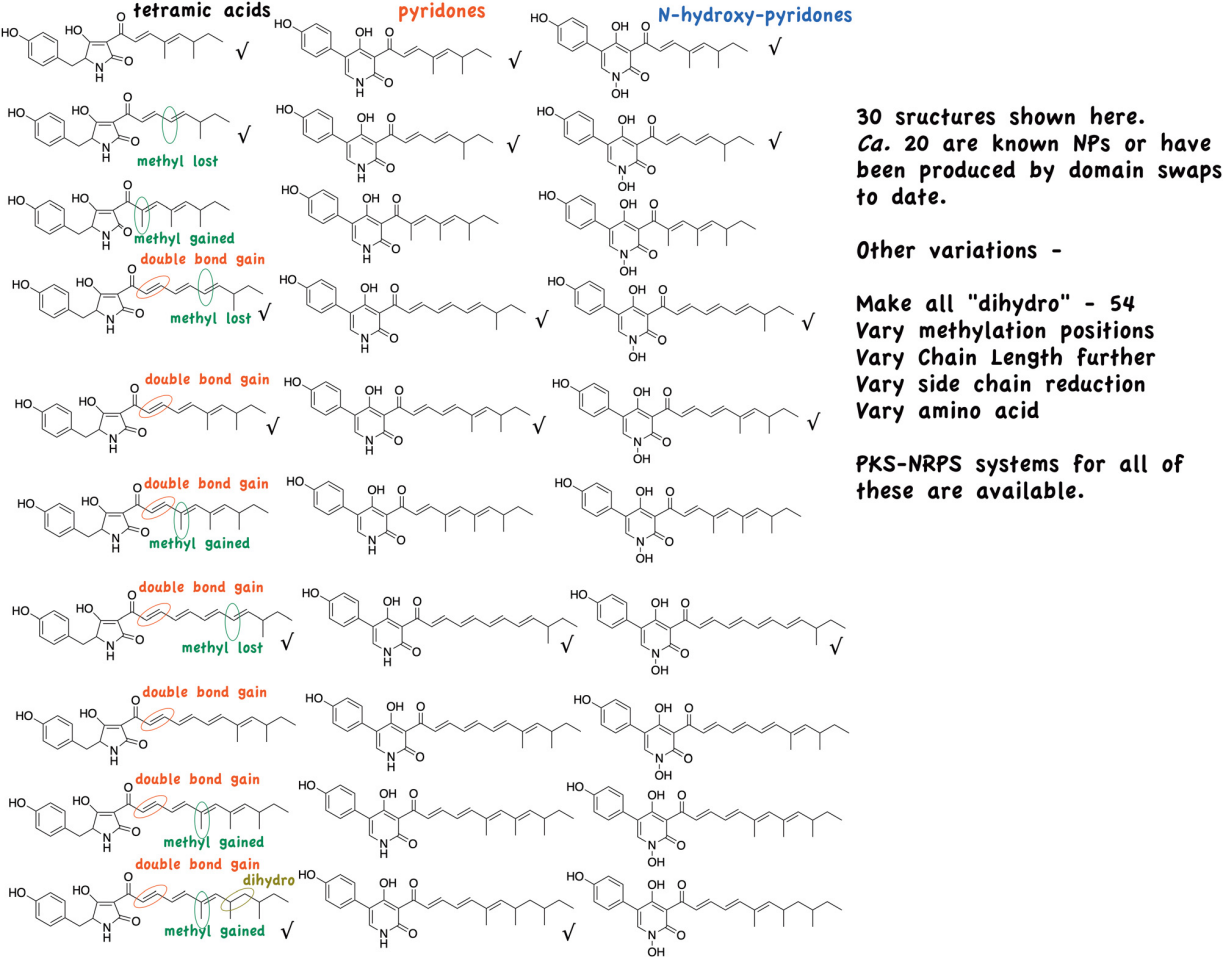
Further products (actual and potential) from tenellin, desmethylbassianin and militarinone domain swaps.

A large variety of these fungal PKS-NRPS compounds differing in polyketide assembly and amino acid component are known and so in principle, these simple domain swaps can potentially give rise to a very large number of novel compounds. As indicted in Fig. 6, many of these have already been achieved.

This ability to not only produce new natural products but also recreate extinct compounds by pathway engineering is a pertinent demonstration of the power of combinatorial biosynthesis as applied to fungal polyketides.

## Cryptosporioptins

**Scheme 4:**
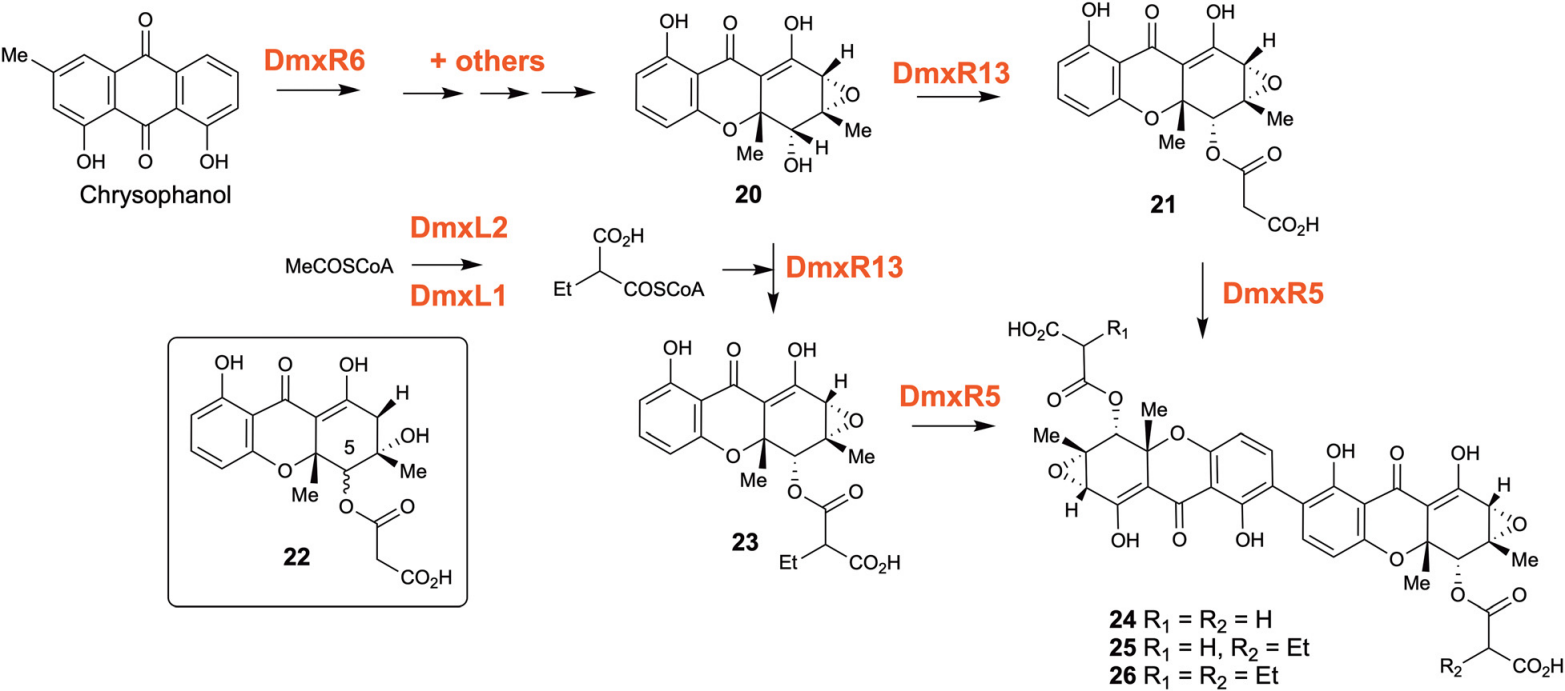
Cryptosporioptin biosynthesis.

A large number of bioactive dimeric xanthones are found in fungi, notably the mycotoxins secalonic acids and related ergochromes, the antimalarial ascherxanthone A and the antimicrobial and anticancer dicerandrol C.^38^ The biosynthesis of these dimeric xanthones have been long studied but uncertainties remain, in particular the timing of the ring cleavage of the precursor anthraquinones,^39^ usually chrysophanol, and the timing of dimerisation of the xanthone monomers.^40^ In the course of studies on the nonadride class of natural products in of *Cryptosporiopsis* sp. 8999,^41^ we isolated three closely related dimeric metabolites and established the structures to be 24–26 which differ only in the presence of ethyl groups on the unusual malonyl substituent.^42^ Genome sequencing identified the cryptosporioptide (*dmx*) BGC, which was confirmed by targeted knockout of the putative cryptosporioptide PKS and subsequent abolition of all cryptosporioptide production. Disruption of *dmxR6*, a putative Baeyer–Villiger monooxygenase gave high titres of chrysophanol as expected, showing that dimerisation does occur after anthraquinone ring cleavage and xanthone formation (Scheme 4). Deletion of *dmxR5* encoding a P_450_ (putative dimerase) abolished dimer production, and two novel metabolites with similar UV spectra to the cryptosporioptides but with molecular weights consistent with monomers were isolated. NMR analysis confirmed that these were hemi-cryptosporioptide A 21 and a shunt product 22 containing a tertiary alcohol at C-6 which was isolated as a mixture of epimers at C-5. When monomer 21 was re-fed to the *ΔdmxPKS* mutant, cryptosporioptide A 24 production was restored confirming this as a pathway intermediate. The *dmx* BGC has a series of unique genes not present in the other dimeric xanthone clusters such as *dmxR2*, *dmxR8, dmxR11-13* and dmxL1-3.

In particular *dmxL2* encodes a highly reducing PKS (hr-PKS) homologous to the lovastatin diketide synthase. The gene *dmxL1* encodes an acyl-CoA carboxylase. *DmxR13* encodes an *O*-acyltransferase, and again this is absent from the other clusters. Disruptions of *dmxL2* and *dmxL1* both abolished production of cryptosprioptide B 25 and C 26, to give exclusively cryptosporioptide A, proving their role in biosynthesis of butyrate and carboxylation to give ethylmalonate and its subsequent incorporation into 25 and 26, presumably by competing with endogenous malonate. It is significant that three different classes of molecule – AQ, monomeric and dimeric xanthones can be accessed by these means. In addition, biosynthetic flux is again redirected to a produce single metabolite from a mixture.

## Conclusions

Natural products isolated from bacteria and fungi have long been an invaluable source of bioactive compounds that have been of immense value in human, animal and plant health, or have served as valuable lead compounds for further development. Limiting issues have been availability in required titres, and in particular their structural complexity making them difficult to access in quantity and to modify their structures by standard synthetic chemistry to enhance activities, stability, solubilities, *etc.* A fundamental rational for biosynthetic studies has always been the idea that if sufficiently well delineated, biosynthetic pathways should be amenable to manipulation to produce analogues and derivatives with enhanced properties. However, achieving this has traditionally been limited to mutagenesis by classical methods, and precursor directed biosynthesis where analogues of intermediates were fed into cultures. With the advent of genome sequencing and computer-based analysis and the normal occurrence in micro-organisms of biosynthetic genes being “clustered” on contiguous stretches of the genome, myriad possibilities for genetic engineering approaches became viable. In this article some of these have been highlighted. These include targeted gene knock-outs to isolate intermediates, selected deletion of genes and addition of genes from other pathways to produce derivatives and analogues, blocking of side or other minor pathways to direct metabolite flux along a single route to avoid separation of mixtures. Heterologous expression of whole pathways in more amenable hosts can allow higher titres and more efficient isolation and purification. The results arising from applications of some of these techniques are illustrated above. Others include mining of genome sequences and switching on “silent” pathways not normally expressed and transfer of whole pathways from non-cultivatable organisms and even expression of environmental DNA. It is an exciting time for this area and after a period when exploitation of natural products became less actively pursued in academia and, in particular, in industry, the study of natural products and their biosynthetic pathways is realising a new and vigorous future.

## Data availability

No primary research results, software or code have been included, and no new data were generated or analyzed as part of this review.

## Conflicts of interest

There are no conflicts to declare.
